# P-932. Retrospective, Multi-Hospital, Quantitative and Qualitative Evaluation of Adding Nirmatrelvir-ritonavir to Formulary

**DOI:** 10.1093/ofid/ofaf695.1135

**Published:** 2026-01-11

**Authors:** Jose Valdes Ledesma, Jefferson Cua, Lourdes R Menendez Alvarado, Erika Dittmar, Timothy Gauthier

**Affiliations:** Baptist Health, Miami, Florida; Baptist Health South Florida, Miami, FL; Baptist Health, Miami, Florida; Baptist Hospital of Miami, Miami, Florida; Baptist Health South Florida, Miami, FL

## Abstract

**Background:**

Nirmatrelvir-ritonavir (NMV/r) and remdesivir (RDV) are conditionally recommended by the Infectious Diseases Society of America for treatment of mild-moderate COVID-19. Use of nirmatrelvir-r in the inpatient setting has been limited due to logistical and regulatory barriers. This study evaluated the impact of NMV/r addition to acute-care formulary following commercial release, exploring both quantitative and qualitative outcomes.

**Methods:**

This was a retrospective, multi-hospital study of patients ≥ 70 years with confirmed COVID-19, who received at least one inpatient dose of either RDV or NMV/r for mild-moderate COVID-19 from March 8 through December 31, 2024. Patients were excluded if they were immunocompromised, had incomplete or missing medical records, or had treatment initiated in the outpatient setting. The quantitative primary outcome was medication cost-avoidance following formulary addition of NMV/r. Qualitative secondary outcomes included progression to severe disease, treatment duration, incidence of adverse events leading to treatment discontinuation, hospital length of stay (LOS), ICU admission within 28 days, 30-day COVID-19-related readmission, and COVID-19-related 28-day all-cause mortality.

**Results:**

Of 215 patients screened, 50 patients received RDV with 159 days of therapy and 50 patients received NMV/r with 116 days of therapy. Demographics are displayed in Table 1. The approximate drug cost without NMV/r would have been $215,000 if RDV was given to all 100 patients. With NMV/r available, actual drug costs totaled $124,000 for RDV and $25,000 for NMV/r, resulting in a medication cost avoidance of $66,000. NMV/r was associated with lower rates of progression to severe disease (11 [22%] vs. 23 [46%], p = 0.02), shorter treatment duration (2 vs. 3 days, p < 0.01), and reduced hospital LOS (2 vs. 4 days, p < 0.01). Other secondary outcomes are listed in Table 2.

**Conclusion:**

Acute-care formulary addition of NMV/r for mild to moderate COVID-19 resulted in substantial medication cost avoidance and was associated with reduced hospital LOS, shorter treatment duration, and lower rates of progression to severe disease.

**Disclosures:**

All Authors: No reported disclosures

